# Wearable Health Devices for Diagnosis Support: Evolution and Future Tendencies

**DOI:** 10.3390/s23031678

**Published:** 2023-02-03

**Authors:** Elena Escobar-Linero, Luis Muñoz-Saavedra, Francisco Luna-Perejón, José Luis Sevillano, Manuel Domínguez-Morales

**Affiliations:** 1 Architecture and Computer Technology Department, ETSII-EPS, University of Seville, 41004 Sevilla, Spain; 2 Robotics and Technology of Computers Laboratory, University of Seville, 41004 Sevilla, Spain; 3 Research Institute of Computer Engineering (I3US), University of Seville, 41004 Sevilla, Spain

**Keywords:** diagnosis aid, wearable, e-Health, physiological signals

## Abstract

The use of wearable devices has increased substantially in recent years. This, together with the rise of telemedicine, has led to the use of these types of devices in the healthcare field. In this work, we carried out a detailed study on the use of these devices (regarding the general trends); we analyzed the research works and devices marketed in the last 10 years. This analysis extracted relevant information on the general trend of use, as well as more specific aspects, such as the use of sensors, communication technologies, and diseases. A comparison was made between the commercial and research aspects linked to wearables in the healthcare field, and upcoming trends were analyzed.

## 1. Introduction

In recent years, there has been an increasing demand for personalized, non-hospitalized healthcare, which has led to the increasing development of telemedicine [1].

Telemedicine can be defined, in general terms, as an integrated system that uses telecommunications and other technologies to exchange health information and provide healthcare services, crossing social, cultural, and geographical barriers [2,3]. This telemedicine allows bidirectional remote contact between patients and medical professionals [4].

The focus of telemedicine shifts to healthcare in different fields, such as the follow-up of acute and chronic infectious diseases, such as hepatitis C virus (HCV) or human immunodeficiency virus (HIV) [5,6], and metabolic pathologies, such as diabetes [4]. It shows efficiency in mental health management, teledermatology, and family doctor consultations [7]. Moreover, the use of telemedicine combined with telemonitoring can be applied as a diagnosis aid system, having the advantage of helping healthcare professionals make early diagnoses of different pathologies, such as diagnostic support systems to help in the detection of possible strokes and myocardial infarction, in the detection of seizures, early cancer, falls, or accidents, among others [8,9,10,11,12]. The use of telemedicine saw higher growth with the advent of the COVID-19 pandemic. Seeking to respect social distancing while maintaining the quality of healthcare, it became even more necessary to use telemedicine for virtual and remote consultations between patients and medical professionals [5].

On the other hand, telemedicine is also a great benefit for patients who do not have easy access to on-site healthcare due to economic or geographic reasons, since it improves accessibility to the healthcare system in a remote way. Thus, telemedicine allows medical care to a larger number of patients, including those in isolated and rural populations [6].

Telemedicine also has economic advantages, as it reduces the costs associated with healthcare. It has been established that the cost of healthcare can be reduced by approximately 56% when telemedicine is practiced [13]. This is also reflected in the patient’s economy, in those countries where healthcare is not public, having a reduction in the cost of treatment, transports, etc. In addition, the use of telemedicine increases patient satisfaction and the uptake of treatment and adherence [6].

In order to practice efficient telemedicine, the remote monitoring of physiological data of patients is essential, and this can be achieved by using wearable devices [5]. Wearable technology in medicine can be defined as electronic instruments that usually contain sensors, microprocessors, and wireless data communications to record medical information in real time [14]. These wearable devices have grown in popularity in recent years thanks to the technological advances in the miniaturization of sensors and other electronic elements [15]. Moreover, in the last decade, the general population is acquiring a healthier lifestyle, which is related to their engagement in using wearables to monitor their physiological signals and fitness achievements [16]. The use of wearables has also increased in the elderly population, as it offers real-time monitoring of potential problems, from critical falls to health complications, enabling early and quick medical action. In addition, wearing these wearables gives them greater confidence in performing tasks independently, and allows their environments to be reassured when they are not around [17].

Originally, wearables in healthcare were oriented toward invasive and implantable devices to monitor cardiac and brain signals. However, nowadays, they are geared toward personal and non-invasive use, being controlled by the users themselves to measure their physiological signals [18,19,20]. Wearables differ from other electronic medical devices in terms of portability, usability, and adaptability [21]. These current wearables are mostly formed by sensors, signal processors, and power supplies [22]. Those sensors have been developed and improved over the years thanks to advances in microtechnology, and they are able to extract clinically relevant information, such as heart rate, blood pressure, body temperature, respiration rate, and body movement [21]. Furthermore, most of these devices also contain the ability to transmit monitored data in real time, integrating communication protocols, such as Bluetooth or Wi-Fi [23].

Overall, wearables can be found in the form of accessories, such as rings or earrings [24], integrated into garments as t-shirts [25] or socks [26], and evenly as tattoos or body parches [27]. However, wearable devices are mostly found in wristbands [28].

Within all of the aspects mentioned above, it is essential that all electronic elements of a wearable device comprise low-power consumption, as this will benefit the device’s battery life and data transmission. Moreover, apart from having a good battery life, it is essential that the device has a minimized weight and size, so that the design of the whole system can be considered as a wearable device. These factors are also important for the user to easily adhere to these devices [29].

In general, the use of wearables in telemedicine has grown rapidly in recent years, bringing many advantages to the healthcare value chain, with benefits in terms of medical personalization, early diagnosis, decision-making, and remote patient monitoring. Although the most widely used commercial wearable devices today are very efficient and offer these advantages, researchers are developing more accurate sensors, looking for new materials and other elements so that smaller and more precise wearable devices can be obtained.

The main goal of this work is to answer the following questions:

(1) What has been the evolution of commercial wearable devices so far?
(2) What has been the evolution of research related to wearable devices in e-health until now?
(3) What is the future trend of commercial wearable devices and how is research on these devices applied to telemedicine?

As mentioned previously, wearable devices are popular in the commercial and research sides, so both cases will be investigated separately. Moreover, as stated above, wearable technology has increased in recent years, so it is necessary to analyze its evolution in-depth and study where it is headed in the future.

In order to answer the first question, we will investigate which were the most popular wearable devices in recent years and analyze their technical specifications. Regarding the second question, the most relevant research works in recent years will be analyzed following certain guidelines. Finally, to answer the third question, it will be necessary to analyze the evolution studied in the two previous cases, comparing the current state of the technologies used both commercially and in research works.

So, this paper will attempt to answer the previous questions through an exhaustive analysis of different commercial wearable devices and the scientific advances in this field. Over the last several years, several reviews of the state-of-the-art wearables applied to different fields of health have been carried out. However, many of them have focused on a specific area of wearable application, for example, to monitor cardiac activity, physical activity, or the deterioration of certain diseases. Furthermore, although other reviews have been carried out focusing on devices marketed in the field of healthcare, the authors have not found in the literature any review that compares the evolution of commercial wearable devices with those investigated, in addition to studying all the aspects related to them, taking into account the technologies they use and the problems they detect, among others. This scoping review aims to fill this gap by comparing the trend of commercial devices with published work.

The rest of the paper is divided as follows: the next section presents the methodology followed in the work. The third section shows the results obtained in the analysis, and in the following section, they are discussed. Finally, the last section presents the conclusions.

## 2. Materials and Methods

**Figure 1:**
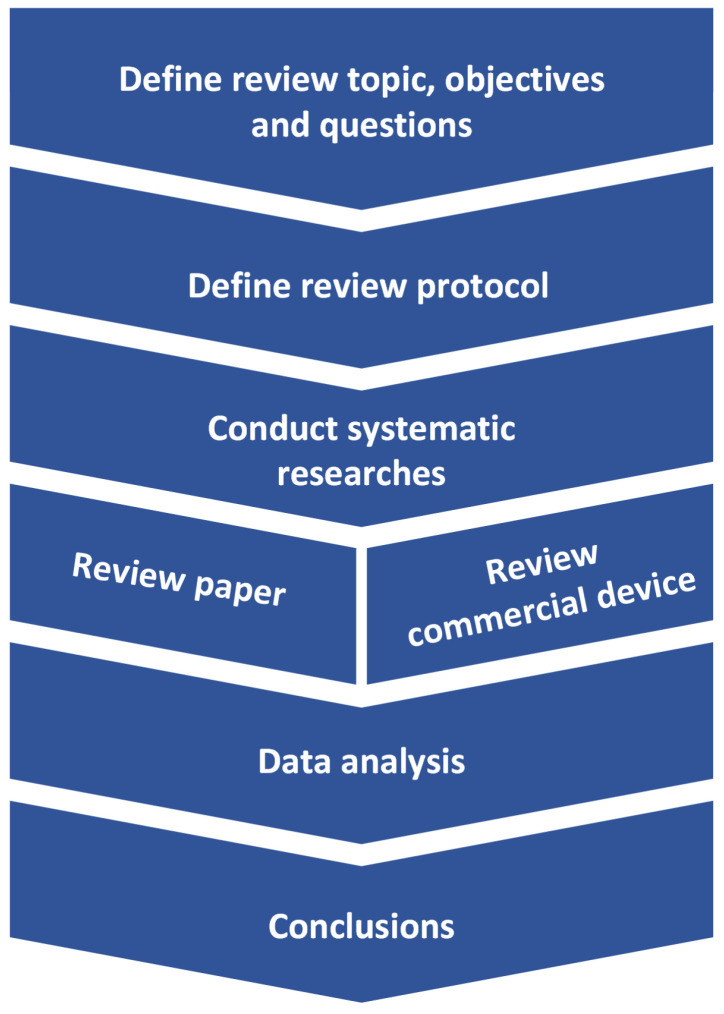
Process followed in this review.

The methodology followed in this review is shown in Figure 1. First, the scope of the review is defined, which in this work consists of wearables in healthcare. Moreover, the questions to be answered in this field and the objectives to be achieved with this review are also defined. After obtaining these initial bases, the protocol to be followed by all the authors for the review is defined and the searching phase can be started. Following these steps, the papers found are reviewed, as well as the wearable devices available on the market, and the relevant information defined in the protocol is extracted. Finally, the questions of the review are answered by analyzing the information obtained by the authors.

The following subsections describe the review protocol in more detail: first, the mechanisms followed to obtain the papers and commercial devices are shown; next, the data extracted from each work and device are detailed and, finally, the process followed to analyze the results is explained.

### 2.1. Searching Phase

Firstly, for the commercial review, the most popular devices in the market from each year were chosen through a Google search. The terms searched were “wearable”, “health”, and “commercial”. The inclusion criteria were all those wearable devices or wearable technologies with functions of monitoring any signal of the human body, focused on the health field. Devices that were not launched to market or were unsuccessful were excluded. Moreover, devices whose technical specifications were not found or were unavailable to the authors were not eligible.

Regarding the literature review, the Scopus search engine was used, as it displays studies from various platforms, collecting papers from more than fifteen different scientific publishers. Moreover, this search engine has an intuitive and functional filtering tool, which makes it possible to filter the papers according to certain keywords and years. Specifically, the combination of keywords searched was as follows: “wearable” AND (“diagnostic support” OR “e-health”). The search was carried out on the titles, abstracts, and keywords of the works.

From the papers identified in the search, scientific studies that used some form of wearable device or technology to measure physiological variables and that are focused on diagnostic support or healthcare were included. Duplicate papers were excluded; papers presented at conferences, pre-prints, and arXiv or bioRxiv works were also discarded. Finally, since we wanted to analyze the trend and progress of wearable technologies and developed devices, papers that presented a scoping review were also excluded.

On the other hand, both reviews were conducted in the last ten years, from 2013 to 2022. Thus, performing the review in the last ten years made it more intuitive, i.e., there was an evolution in the different technologies and devices used.

**Figure 2:**
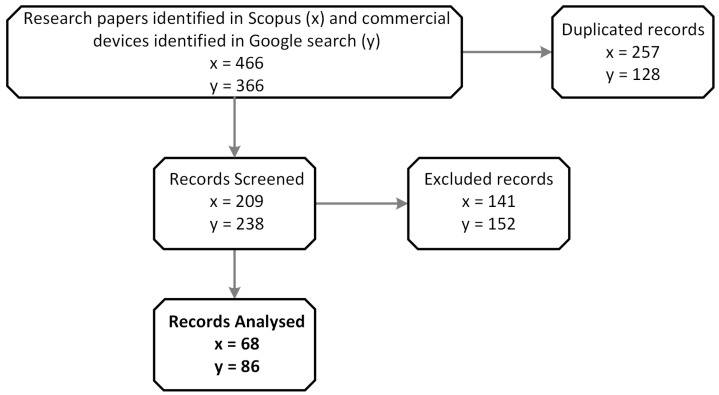
Searching phase results.

The results of both search processes are shown in Figure 2.

In summary, 466 scientific papers were initially obtained, of which 257 studies were excluded because they were duplicates or did not meet the inclusion criteria and, of these, a further 141 papers were excluded because they were scoping reviews, conference papers, or preprints. Finally, of these, 141 works did not meet the initial criteria, their content was not relevant to this review, or they did not use a wearable device. Thus, 68 papers were analyzed exhaustively in this review.

On the other hand, in the search process of commercial devices, 342 initial results were obtained, of which, 86 devices were finally analyzed; the remaining ones were either duplicated, not marketed, or their technical specifications were not found.

### 2.2. Extracted Information

Once the searching phase is completed, the next step consists in extracting relevant information about the commercial wearables and the scientific papers. Thus, from these data, an exhaustive analysis may be carried out and the initial answers of this review may be answered. The information extracted from both types of records will be slightly different (because for commercial wearables, information about the market and companies can be extracted); while for published works, information about the journal or the number of citations can be obtained. However, for both reviews, some items extracted will be the same.

Firstly, for each commercial wearable, the following information will be extracted:

- Name: commercial name of the wearable device.
- Company: name of the manufacturing company.
- Year: year of the market launch of the wearable.
- Country: the country where the wearable was produced.

Regarding the literature review, the details obtained from the studies are listed below:

- Title: title of the scientific work analyzed.
- Authors: name of the authors of the manuscript.
- Year: publication year of the analyzed study.
- Cites: number of citations received up to the date of this review.
- Journal: scientific magazine where the work was published.
- Country: country of the localization of the first author during the research.

Lastly, the following information will be extracted commonly in both commercial review and literature review:

- Wearable type: category of the wearable analyzed, which can be a wristband, a watch, a garment, or motes, among others.
- Communication protocol: name of the communication protocol used in the device to send the registered data.
- Sensors: wearable sensors used to monitor physiological signals and other body parameters.
- Focus target: main physical and physiological features detected by the device.

After extracting this information, the analysis of global and individual wearables can be carried out. This process is explained in the following subsection.

### 2.3. Analysis Process

In order to carry out an exhaustive analysis of the information extracted from each device found on the market and in the literature, the results obtained year after year will be taken into account. Thus, it will be possible to evaluate the evolution of the different items analyzed over the ten years of this review. On the other hand, the information extracted will also be evaluated globally, grouping each of the characteristics analyzed regardless of the year of the work. After obtaining these analyses, it will be possible to answer the questions introduced in this review.

Firstly, the number of commercial devices analyzed each year will be shown, as well as the number of published papers reviewed year by year. Therefore, the evolution of wearables on the market and in the literature may be assessed.

Next, the distribution of the regions in which commercial wearables were developed and where research was carried out will be studied. Thus, we will evaluate the distributions according to continents and countries. In the case of content distribution, the analysis will also be carried out for each year, looking at the evolution of each continent’s work over the years. In both cases, as in the previous case, the results will be shown firstly for the commercial wearables reviewed and then for the literature review.

The remaining analyses will consist of assessing the global distribution of wearable types for both reviews. The same procedure will be followed for the methods used for the transmission of the recorded data and the sensors used. In this last case, the analysis of the annual evolution will also be shown. Finally, the characteristics detected by the devices in both cases will also be analyzed.

With these evaluations, the results can be discussed, allowing answers to future trends in wearable research and commercialization, as well as their evolution so far in the fields of health and diagnostic support.

## 3. Results and Discussion

In this section, the results obtained following the analysis process detailed above will be shown. First, the evolution of the commercial devices launched in the market and the evolution of the manuscripts published will be analyzed. This will be done regarding the records screened and also according to the total works published in the literature. Next, an analysis of the devices on the market and the works published in each region will be carried out. Then, the results of the most developed type of wearables will be shown, as well as the communication protocols they integrate and the sensors that form them. This will be exposed at the same time for the review of commercial devices and the review of scientific studies. Following the same process, the main targets measured by the devices will also be analyzed.

### 3.1. Wearables Evolution

In order to observe the global evolution of wearable technology, Table 1 shows a summary of the number of studies published each year since 2000. These are the results obtained for wearable and health research studies, taking into account the total publications, not only those considered for the analysis of this review. As can be seen, there has been a clear increase in the number of papers published over the last 22 years, from 616 studies on wearables and health in 2000 to more than 52,000 in the last year. What is most striking is that every year has seen a positive variation in publications compared to the previous year, which indicates the popularity of this field and the continuous advances in the development of wearable devices for health. However, the same case is not observed for the last two years, with a drop in the number of papers published in 2021 and 2022. The reason for this is probably the restrictions experienced during the global pandemic. This effect is seen in more detail in the analysis of the evolution of the papers analyzed in this review.

**Figure 3:**
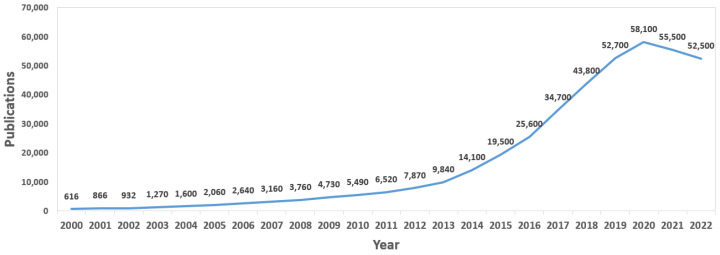
Global evolution of journal publications.

The same results shown in Table 1 can also be seen graphically represented in Figure 3. The evolution in the number of works published each year is clearer; there has been very pronounced growth since 2013 (reaching a peak in 2019). A drop in the number of published papers is observed.

With this overall publication evolution, the growing trend of this field is seen more clearly. Next, the evolution of both the commercial wearables launched in the market and the scientific works published is detailed.

**Figure 4:**
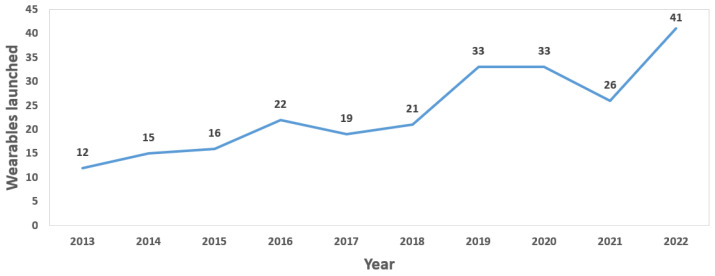
Evolution of commercial wearables launched in the market.

As can be seen in Figure 4, the evolution of marketed wearable devices has followed an upward trend since 2013, suffering a slight recession in the years 2020 and 2021 due, presumably, to the global pandemic; however, this trend can be seen to return to its usual course in the year 2022.

**Figure 5:**
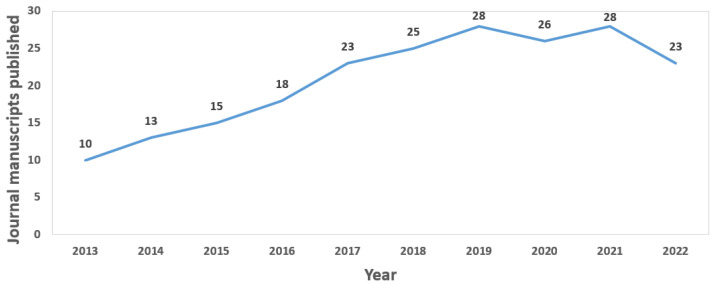
Evolution of journal publications.

On the other hand, Figure 5 shows the trend in the use of wearable devices in journal articles. In this case, the trend is more linear, again suffering stagnation in the years 2020 and 2021, which, again, may be due to the circumstances arising from the pandemic. Although it is true that research has boosted during the pandemic years (and significant increases in publications were observed), the same is not true for the problem studied in this paper; since, in order to publish an article related to the use of wearables, it was previously necessary to be able to evaluate it with a set of participants and, due to the pandemic, testing with people has been drastically reduced. Finally, the year 2022 also represents a drop in publications (one month before the end of the year, which is when the statistics were extracted), so it may simply be the result of not having finished the year, or also due to the restrictions on testing devices with people (which have continued to be present in the first half of 2022 in many countries).

Moreover, by comparing commercial wearables with research in this field, it can be observed that after the start of a global pandemic, commercial devices have returned to their previous trend, even growing further. This is probably thanks to the inclusion of new sensors in those devices, which will be studied in the following sections. This effect may influence the number of publications in the following years, since with the introduction of new commercial sensors, the number of research papers may increase again. Thus, by using new types of sensors in commercial devices and by reducing the restrictions due to COVID-19, it is expected that the evolution of publications will grow again in following years. This will have to be studied in future reviews.

Table 2 lists the number of selected commercial devices each year, identifying the manufacturer, and Table 3 lists the most used commercial devices according to the producing company. On the other hand, Table 4 lists the selected papers for each year after the filtering process.

After observing the general evolution over the last ten years, some more specific aspects of the published works and marketed devices will be analyzed.

### 3.2. General Data Analysis

The aspects to be analyzed independently are the countries of commercialization or origin of the authors of the publications, the type of wearables, the communication protocols used, the sensors integrated into the wearables, and the focus target.

#### 3.2.1. Countries

**Figure 6:**
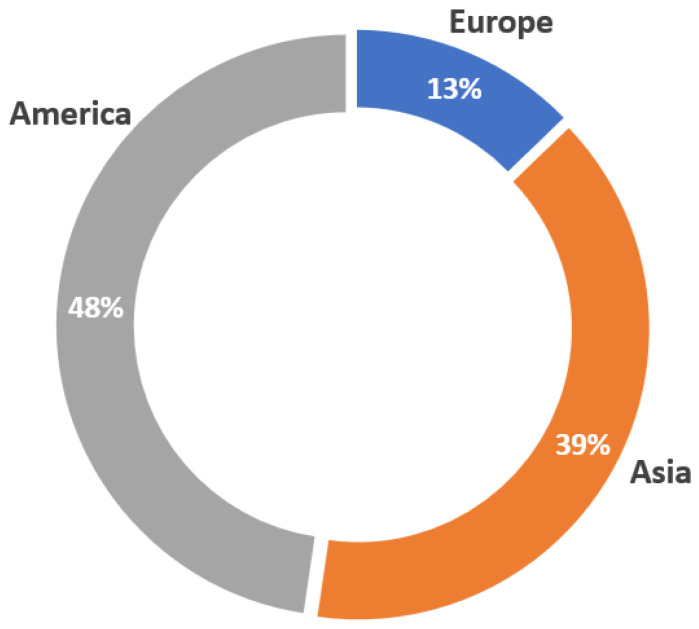
Location distribution for the commercial devices marketed.

This first part focuses on where the commercial devices analyzed were marketed. Figure 6 shows the distribution of the continents where the reviewed wearables were developed. America leads this distribution, with 48% of the wearable devices launched to the market. It is worth noting that the regions established for each device correspond to the location where the headquarters of the company that developed the wearable is settled. The second leading continent, in this case, is Asia, with 39% of the devices analyzed. Finally, it can be seen that the distribution of devices created in the European Union (EU) decreases significantly, with 13% of wearables developed in this region.

**Figure 7:**
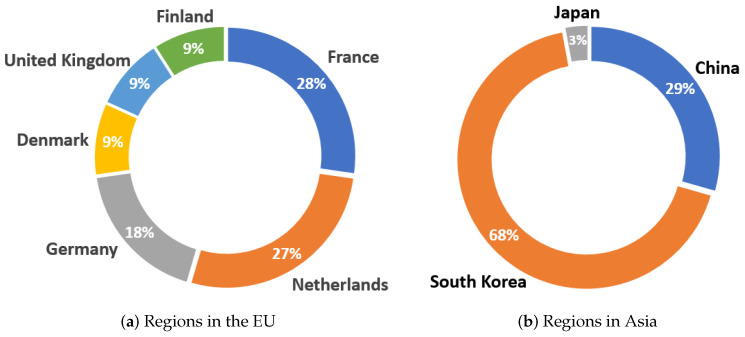
Country distribution for the commercial wearables developed in Europe (**a**) and Asia (**b**).

Together with the analysis of the distribution by continent, Figure 7 shows the countries’ division within each continent. In the case of America, which is the continent with the highest number of devices marketed, only one device was created in Canada, while 98% of the remaining wearables correspond to the United States of America (USA). This distribution is not shown graphically. Figure 7a shows the countries among which devices marketed in the EU are distributed. Moreover, 28% of the devices are developed in France, corresponding to 4% of the total. This is the European country with the most devices launched in the market, together with the Netherlands, which contains 27% of the European devices. The third country in this region that developed the most wearables was Germany, with 2% of the total devices. Finally, three countries developed only one wearable device out of the total, i.e., Denmark, UK, and Finland, with 9% of the European distribution (1% of the total distribution).

On the other hand, Figure 7b shows the distribution of countries in the Asian region. In this case, the first place goes to South Korea, having marketed 68% of the devices, corresponding to 27% of the total. This is followed by China, where 23 of the 86 wearables analyzed in this review were created. Finally, Japan accounted for 3% of the Asian distribution.

**Figure 8:**
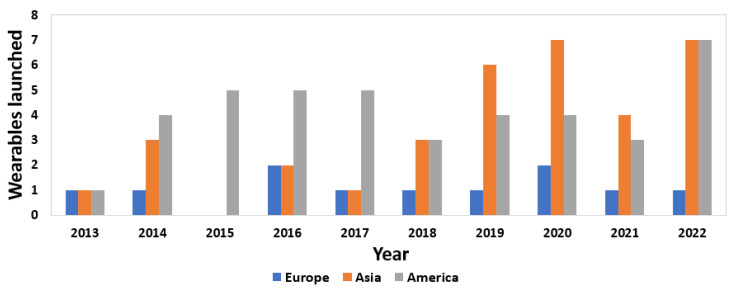
Location distribution of the developed commercial wearables by year.

After the global analysis of the distribution of the regions in which commercial devices were developed, Figure 8 shows the same distribution by continent, but this time considering the prevalent regions in each year. Thus, it is possible to see the evolution of the commercialization of wearables in different locations each year. In 2015, only devices developed in America were marketed, which may be influenced by the launch of the first Apple Watch models. On the other hand, from 2014, the number of devices launched in America remained constant until 2017, when the number decreased again and the Asian region took over. The number of devices developed in Asia showed high growth until 2020. However, in 2022, the number of devices marketed in America and Asia remained equal. On the other hand, in the case of the EU, there were few wearables launched annually. In 2013, all three continents launched only one device each.

**Figure 9:**
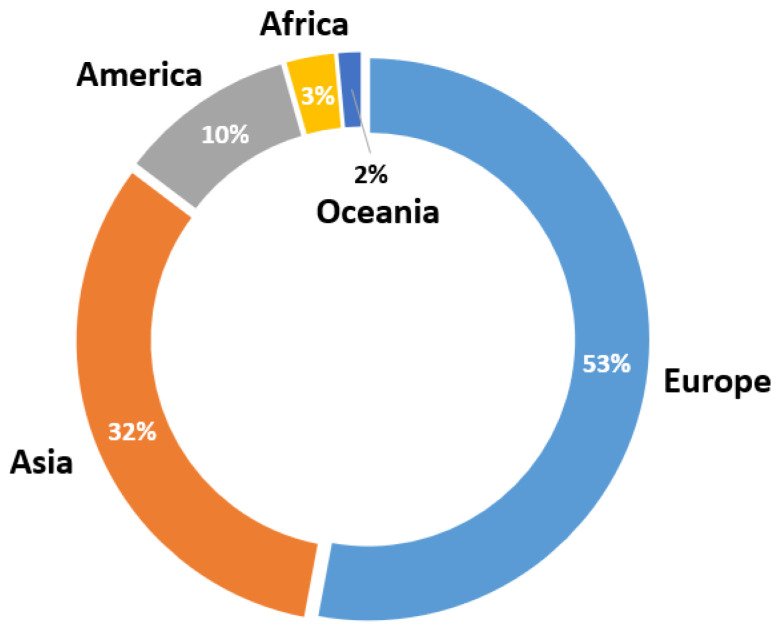
Location distribution for the published journal manuscripts.

Following the same process, we also analyzed the locations of the manuscripts published in journals. Figure 9 shows that the region with the largest number of papers published in the last ten years was the European Union, with slightly more than half of the total papers, more specifically 53%. The second place in this distribution goes to the Asian continent, with 32% of the total number of scientific papers. In third place is America, having published 10% of the total papers. Finally, a low proportion of all papers were published by authors from Africa and Oceania.

**Figure 10:**
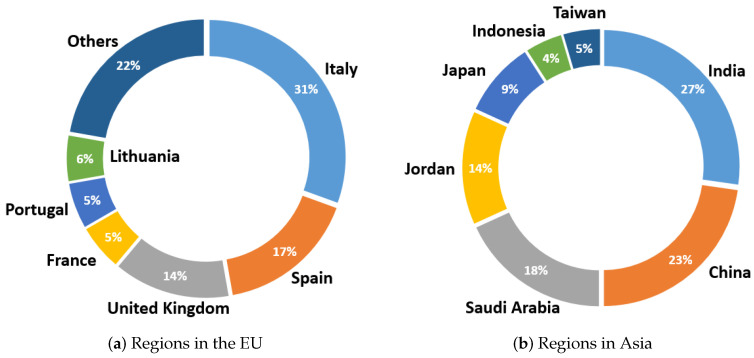
Country distribution of published journal manuscripts in Europe (**a**) and Asia (**b**).

For the regions that published the most papers, the distribution by country can be seen in greater detail in Figure 10. In the case of the EU (see Figure 10a), the majority of papers published on wearables in healthcare and diagnostic support were in Italy, with 31% of the European papers published (17% of the total papers). This was followed by 17% of published manuscripts developed in Spain and 14% in the United Kingdom (UK). Studies from France, Lithuania, and Portugal were also included. Finally, other countries also made contributions to research on wearables in healthcare. In this case, 22% of the European works correspond to Switzerland, Cyprus, the Netherlands, Slovakia, Poland, Hungary and Sweden, each with one work published in a journal.

Looking at the distributions by Asian countries (Figure 10b), India is in the lead, followed by China and Saudi Arabia, with 27%, 23%, and 18% of the papers published by Asian authors, respectively. Jordan also contributed a significant number of papers on wearables, with 14% (corresponding to 4.5% of the total). Finally, other Asian countries that developed research papers in this field were Jordan, Indonesia, and Taiwan, although all to a lesser extent.

**Figure 11:**
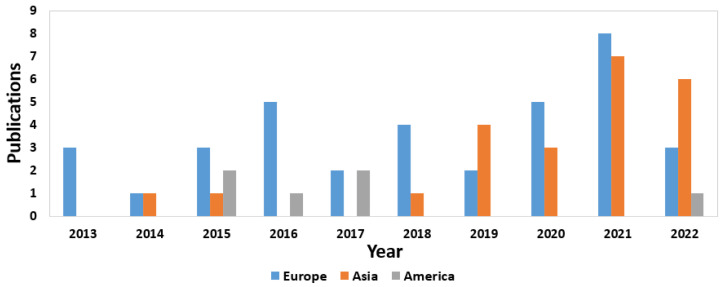
Location distribution for the published journal manuscripts by year.

On the other hand, as in the analysis of commercial devices, the annual evolution of the published papers from each continent is also shown here, taking into account those that obtained the highest number of publications at the global level. As seen in Figure 11, throughout the ten years of the review, the EU had several significant contributions each year, reaching its first peak in 2016; in the following years, it dropped slightly, until reaching another maximum number of published manuscripts in 2021. However, in 2022, the trend changed and the Asian continent had more contributions. This effect also occurred in 2019, when publications from Asia started to increase (up until 2018, only one was recorded in some years, while in others—none). Finally, the low number of publications contributed by America can be verified (only having registered papers in four out of the ten years analyzed). Overall, the leadership of Europe and Asia in the number of publications was evident, although in the last year, the Asian continent contributed twice as many papers as the EU.

#### 3.2.2. Type of Wearables

Within the analyses, the different types of devices that include the reviewed wearables have been included. These technologies can consist of devices placed on the wrist, such as smartwatches or wristbands; they can also be integrated into different garments, such as socks, T-shirts, or shoes; or they can be directly motes of sensors distributed over different parts of the body, forming wireless body area sensor networks (WBSNs). They can also consist of accessories, such as rings, earrings, or headsets, among others [14].

**Figure 12:**
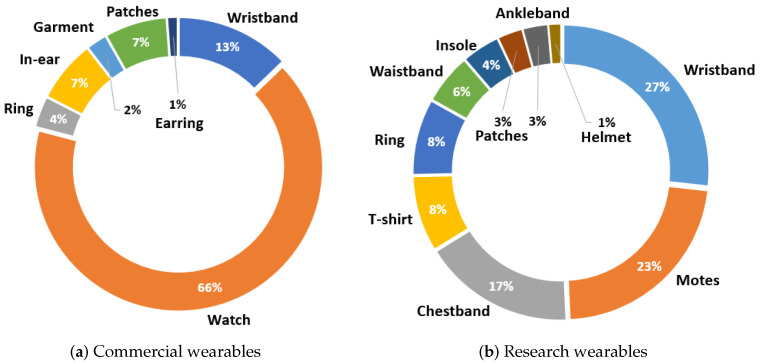
Distribution of the type of wearable devices marketed (**a**) and the type of wearables used in the scientific works (**b**).

Figure 12 shows firstly the overall distribution of the types of wearables analyzed in the market size, and then the distribution of the types of wearables developed in the literature. In the first case, it can be seen that the vast majority of wearable devices used in health and that were commercialized consist of watches (see Figure 12a). Specifically, watches account for 66% of the total devices analyzed. To a lower extent, 13% of the wearables are wristbands. On the other hand, other devices marketed correspond to body patches and wearables placed on the ear, such as earphones. Each of these types corresponds to 7% of the total number of devices on the market. Finally, a few wearables consist of rings, garments, and earrings, accounting for 4%, 2%, and 1% of the total, respectively.

On the other hand, Figure 12b shows the same analysis for the devices developed in the manuscripts published in journals. In this case, the majority of devices are divided between wristbands (26%) and motes (22%). In addition to these types of wearables, there are also chest bands (being 17% of the total). Compared to commercial wearables, in this distribution, there is more variety in the type of wearables developed. Thus, wearables such as T-shirts, rings, waistbands, and insoles have also been integrated, each making up 8% to 4% of the total. Finally, a very small number have also been developed in the form of patches, ankle bands, and helmets.

#### 3.2.3. Communication Protocols

**Figure 13:**
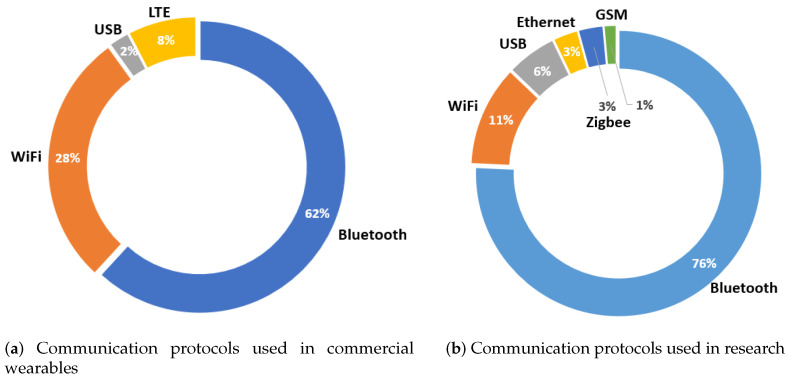
Distribution of the communication protocols integrated into the marketed devices (**a**) and those developed in the literature (**b**).

Following the analysis, Figure 13 shows the results of the distribution of the different communication protocols integrated into the wearables. First, it can be observed that for the wearables brought to the market (see Figure 13a), 62% of the total devices transmit physiological data via Bluetooth connection. On the other hand, 28% integrate the ability to send the monitored information via Wi-Fi. Finally, some commercial devices also integrate long-term evolution (LTE) connection (8%), while very few send the collected data via USB.

Observing the same trend in research devices (see Figure 13b), the majority of wearables integrate Bluetooth communication (76% of the total). The second place is also taken by Wi-Fi communication, which is embedded in 11% of the total devices. On the other hand, 6% of the wearables developed in the literature transmit their data via USB. Finally, in this case, different communication protocols integrated into a few devices can be observed, such as Ethernet, Zigbee, and (to a lesser extent) the global system for mobile communications (GSM).

#### 3.2.4. Sensors

**Figure 14:**
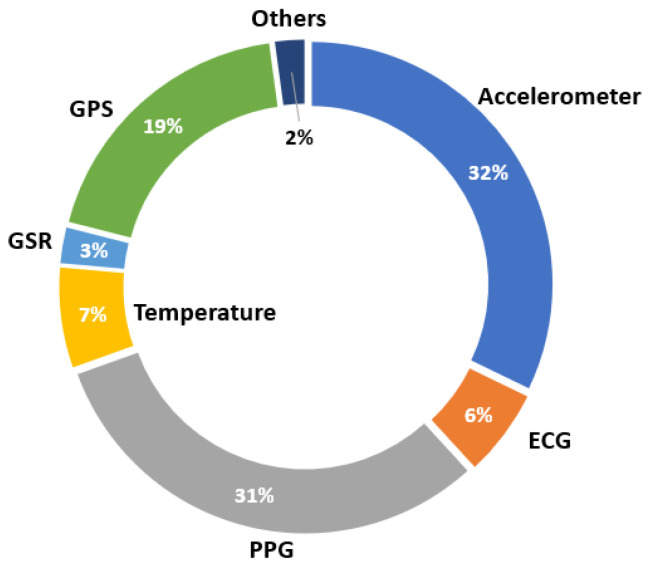
Distribution of sensors used in marketed wearables.

This section analyses both types of reviews according to the different sensors that are integrated into wearable systems. Firstly, Figure 14 shows the distribution of the sensors used in the devices on the market. In this case, accelerometers and photoplethysmographs (PPGs) are the most widely used sensors at the commercial level, both being integrated into 30% of all wearables. The photoplethysmograph is based on a light beam that measures the change in blood volume and, with it, the heart rate can be calculated. Blood pressure can also be calculated with these sensors in some cases. On the other hand, the third most frequently used component in wearables launched on the market is GPS, being on the 18% of all devices. With this component, wearables are able to capture the user’s location and, together with an accelerometer, can perform detections related to location and body position. On the other hand, temperature sensors and electrocardiography (ECG) sensors are also used in 7% and 6% of total wearables, respectively. Finally, a smaller number of devices contain galvanic skin response (GSR) sensors, being present in 3% of the devices. The GSR sensor measures the electrodermal activity (EDA) and skin conductance, whose variations are produced by the sweating of the human body, which can be related to certain emotions. Finally, 2% of the sensors that make up the devices on the market are of a different type. More precisely, they consist of electromyography (EMG) sensors, blood pressure sensors, and glucose level sensors.

**Figure 15:**
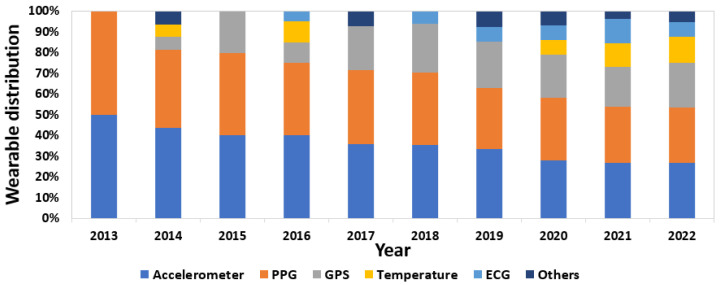
Distribution of sensors used in marketed wearables by year.

In addition to this, Figure 15 shows the distribution of sensors used each year. It can be seen that in the first year of this review, only devices with accelerometers and PPGs were marketed. However, the trend moves toward greater use of other types of sensors, with four more sensors in 2022 compared to the first year. This effect produces a balance in the distribution between accelerometers, PPG, GPS, and other sensors. From 2014, temperature sensors began to be used; from 2016, ECG sensors bean to be integrated into wearables. This change seemed to settle down from 2020, when both were integrated into existing devices and their distribution increased slightly.

**Figure 16:**
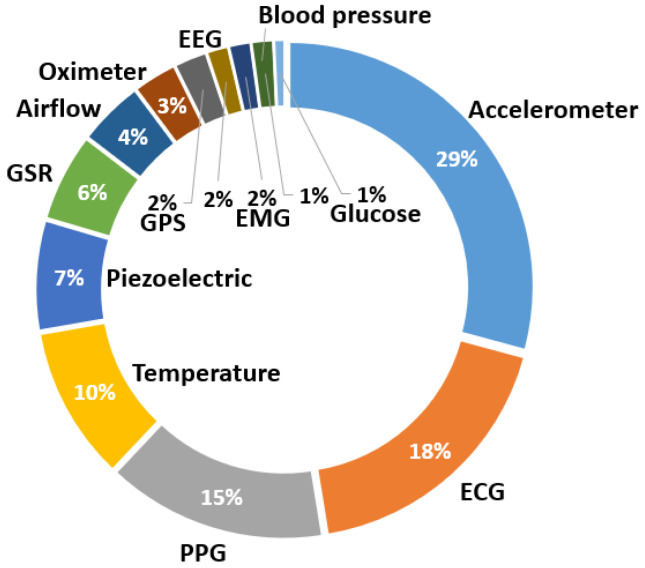
Distribution of sensors used in published manuscripts.

Regarding the review of published works, the same analysis process is followed. Figure 16 shows the global distribution of the sensors used. In this case, accelerometers continued to lead, being present in 29% of the devices developed in the research. However, in this case, the second most used sensor was the ECG, closely followed by the PPG, being integrated into 18% and 15% of the wearables in the literature, respectively. Temperature sensors were also used in 10% of the cases, piezoelectric sensors in 7% of the devices, and GSR sensors in 6%. Finally, other sensors were also investigated, but in fewer works, such as air sensors, oximeters, GPS, electroencephalography (EEG) sensors, EMG sensors, blood pressure sensors, and glucose level sensors.

**Figure 17:**
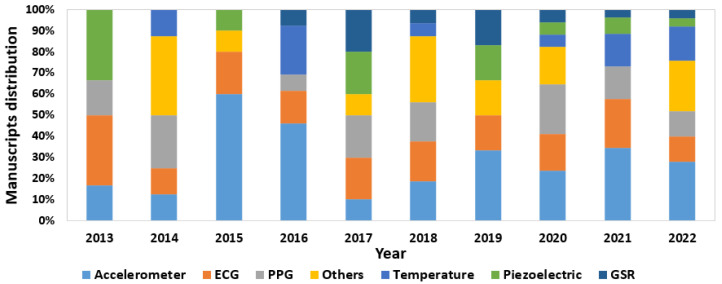
Distribution of sensors used in published manuscripts by year.

For the annual evolution analysis (Figure 17), it can be seen that—in all of the years—studies used accelerometers, ECGs, and PPGs. It is interesting to note that, although the use of accelerometers prevail, from 2017 onward, the use of other types of sensors was incorporated, until reaching the last years, especially 2020 and 2022, where all types of sensors were investigated in different wearables, showing the possible trend of using new types of sensors.

#### 3.2.5. Focus Target

**Figure 18:**
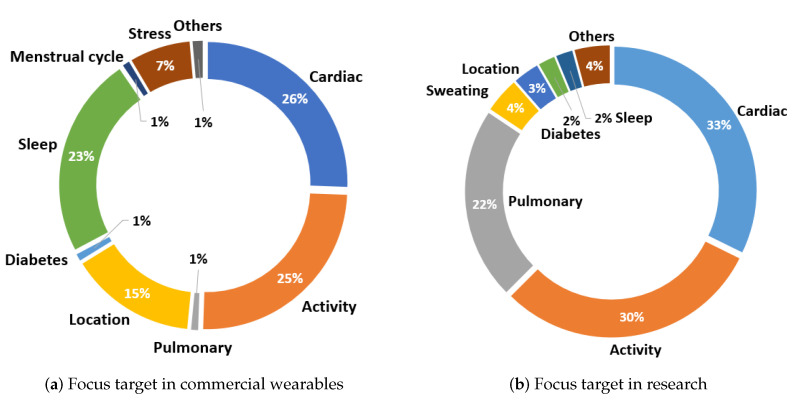
Focus target of the works found in the marketed devices (**a**) and those developed in the literature (**b**).

The last part of this analysis corresponds to the distribution of the events detected using commercial wearables as well as devices developed in the literature. This part is closely related to the previous analysis since depending on the type of sensors used, one type of problem or another will be detected. It might be intuitive to think that with certain sensors the type of monitoring that will be done is obvious; for example, it is logical to think that with a GSR sensor, the level of sweating is measured; however, through this sensor, it is also possible to obtain stress levels, which is necessary to specify. Thus, Figure 18 shows the distribution of each measured problem in general terms.

In terms of commercial devices (Figure 18a), the activity target, corresponding to 25% of wearables, including body movement detection, fall detection derived from the previous measurement, accident detection, step measurement, and distance traveled. Within the cardiac target are heart rate detections, arrhythmia detections, blood pressure, blood oxygen saturation measurement, and caloric expenditure detections. These measurements are integrated into 26% of commercial devices. In 23% of all devices, sleep phases are detected, and in 15% of devices, the user locations are measured. Stress measurements are present in 7% of wearables. On the other hand, few devices (1%) integrate the detection of breathing-related events, diabetes detection, and menstrual cycle status. Finally, the other detection labels include mental health monitoring, epilepsy, and sweating.

On the other hand, Figure 18b shows the targets that the wearables developed in the published manuscripts focus on. In this case, the most common detections are related to cardiac events, followed by user activity monitoring and pulmonary event detections. Each of these is integrated into 33%, 30%, and 22% of the papers, respectively. Pulmonary target refers to cough detections and respiration rate measurement. Sweating measurements are used in 4% of the devices in the literature, locations of users are detected in 3%, diabetes is detected in 2%, and sleep phases in 2%. Finally, in this case, the other detections include emotion measurements and epilepsy detections.

Finally, if we compare the evolution of the use of wearables in the research and commercial fields, and compare them with Gartner’s Hype Cycle for wearable devices (detailed in https://www.gartner.com/en/newsroom/press-releases/2021-01-11-gartner-forecasts-global-spending-on-wearable-devices-to-total-81-5-billion-in-2021 (accessed on 1 December 2022)), we can find some interesting aspects:

- All topics related to smartwatches and wristbands were considered in the first study regarding wearable technology (in 2016: https://betakit.com/11-predictions-for-wearable-tech-in-2016/ (accessed on 1 December 2022)) as aspects to be taken into account in the next 2 to 5 years (until 2021, when a second study has confirmed it). This can be clearly seen in the trend that both commercial devices and research studies have undergone in this study.
- Those wearables located in less common areas (such as shoes or head) or those sensors less common at the beginning (such as sweat sensors or electromyography) appeared as aspects to be taken into account in the next 5 to 10 years (that is, from 2021 to 2026). This aspect seems to be fulfilled also at present, as GSR sensors are starting to be used now (the first works are from 2018 to 2019 in the research field, with promising results), while in commercial devices, they were not integrated until 2021.
- Wearables that are uncommon today, such as glucose meters, UV monitors, or analysis patches, are difficult to find today except in some preliminary research work. This is equally in line with what was indicated in the first Gartner study, as it estimated a time of more than 10 years to reach its peak (around 2026), and continues with this tendency in the recent study.

However, other aspects that were predicted at the beginning study that did not come into fruition (or did not coincide temporally) in the last study ( that can be checked in this work) are as follows:

- Gait analysis: While estimating that it would peak more than 10 years after the study (this is after 2016), research papers found since 2017 focused on this aspect, in addition to multiple commercial devices that can be found today in both the medical and fitness fields. Therefore, this trend has been brought forward.
- Smart Rings: With these types of wearables, something similar happens as with the previous technology. It was predicted that their use would intensify much later than it has, since commercial solutions that extract physiological information from rings are now readily available. However, the “smart” aspect may continue to evolve in subsequent years.

Other aspects that we cannot determine if they reached their peak or not are as follows:

- Exoskeleton: Gartner’s 2016 study indicated that the peak of this technology would be reached in more than 10 years. Currently, multiple research papers related to exoskeletons can be found, as well as some non-affordable commercial solutions. Perhaps, this technology should evolve sufficiently to lower costs and become more accessible to the general population, and it will be then when it will presumably reach its peak.
- Smart Garments: This last aspect also presents many unknowns, since the adjective “smart” is very ambiguous and can imply anything from a simple warning or detection of activity to an in-depth analysis of the information. In the latter case, there are several research studies focused on integrating AI algorithms in wearables, but due to the computational requirements they need, we still cannot find anything similar at a commercial level (everything commercial is focused on transmitting and processing in a mobile device or the cloud). That is why, for this topic, there is still a long way to go, and it may be several years away from reaching its peak of interest.

## 4. Conclusions

In this work, we present a detailed analysis of the use of wearables in the field of health, presenting the devices marketed in the last 10 years, as well as the research work on the subject. In general terms, it can be observed that the research field has suffered considerably during the years of the pandemic, but not the commercial field, which has seen an upturn in the last year.

If we focus on more specific aspects, it is important to highlight the duality existing between Europe and America: while Europe has a great advantage in research works (with America being the third continent after Asia), America tops the list in the field of commercialized devices (with Europe now being third after Asia). In both cases, Asia denotes a stable second position in both aspects. It is remarkable that the continent that launches the most commercial devices per year produces hardly any research work. The opposite effect is true for the continent that does the most research, as it barely markets any devices. However, the Asian continent is the most stable in this respect, especially in recent years, with a balance between published work and marketed devices.

Regarding wearables, one interesting aspect to take into account is the rise of smartwatches in the commercial field. In this research field, it is not common to use smartwatches, since their functionalities exceed the needs of the studies, and that is why wristbands, motes, and chest bands are commonly used. However, commercially speaking, there is a need to adapt these devices to make them more usable, and that is where smartwatches come in: since the users are used to wearing watches, it is not an impediment to add more functionalities to them; thus, sensors that are usually used in research in other parts of the body (such as accelerometers in motes or waistbands, or heart rate sensors in chest bands), are integrated into smartwatches. While it is true that these sensors provide more reliable information in the original locations, for the end user, a usable device is preferable even if it has a higher margin of error (a margin that is being reduced each year thanks to advances in technology).

Next, regarding the communication technologies and the sensors integrated into the wearables, there are several similarities between commercial and research devices: Bluetooth is the dominant communication mechanism in all cases (although Wi-Fi is an alternative to be taken into account in marketable devices focused on home monitoring), and the most commonly used sensors in both cases are those for detecting physical activity/events linked to movements (mainly accelerometer) and those designed to obtain cardiac information (ECG and PPG, although in marketed devices, the PPG predominates due to the difficulty in locating the three probes that make up the ECG).

This last aspect of the sensors also marks the similarity in terms of the focus target of the wearables, which are mainly focused on detecting heart problems and physical activity.

If we unify the partial analyses included in the previous section, we can conclude that the trend in the coming years is to use wearable devices placed on the wrist (wristbands and smartwatches) with sensors that provide physiological information, with the PPG sensor and sweat sensor gaining special importance. The use of sensors that must be located in other positions on the body (such as EEG, EMG, or ECG) is ruled out, as it is clear from the commercial evolution that the wearables with the longest history are precisely those that are most usable and comfortable for the user. Finally, there is a trend in the analysis of gait-related aspects, but the use of wearables in sports shoes seems to be, for now, limited to fitness aspects.

In summary, analyzing the trend in the use of wearable devices, it seems that the next few years will be essential for the integration of smart technologies in the world of wearables. It is, therefore, very important that the technology evolves sufficiently so that complex processing algorithms can be integrated into the wearables themselves (without the need to make up for this lack with a smartphone), and be able to perform real-time detections and classifications in the wearables themselves.

## Figures and Tables

**Table 1:** Global evolution of journal publications.

Year	Number of Publications	Variation
2000	616	-
2001	866	250
2002	932	66
2003	1270	338
2004	1600	330
2005	2060	460
2006	2640	580
2007	3160	520
2008	3760	600
2009	4730	970
2010	5490	760
2011	6520	1030
2012	7870	1350
2013	9840	1970
2014	14,100	4260
2015	19,500	5400
2016	25,600	6100
2017	34,700	9100
2018	43,800	9100
2019	52,700	8900
2020	58,100	5400
2021	55,500	−2600
2022	52,500	−3000

**Table 2:** Commercialized devices selected for this study, ordered by publication year.

Year	#	Manufacturer (Devices)
2013	3	Fitbit, Iriver, Withings
2014	8	Empatica, Fitbit, FreeWavz, Garmin, Jabra, LG, Samsung(2)
2015	5	Apple, Fitbit, Garmin, Lumafit, Owlet
2016	9	Amazfit, Apple(2), Cossinus, Fitbit, iRythm, Philips, Samsung, Siren
2017	7	Apple, Fitbit, Garmin, Joule, Philips, Speac, Xiaomi
2018	7	Amazfit, Apple, Diamontech, Fitbit, Matrix, Samsung, Xiaomi
2019	11	Amazfit(3), Apple, Fitbit(2), Garmin, Omron, Philips, Samsung, Sugarbeat
2020	13	Amazfit(5), Apple, Empatica, Fitbit(2), Philips, Samsung, Withings, Xiaomi
2021	8	Amazfit(2), Apple, Garmin, Oura, Philips, Samsung, Xiaomi
2022	15	Amazfit(4), Apple(2), Bodimetrics, Circular, Fitbit(2), Garmin(2), Samsung, Xiaomi(2)
**TOTAL**	**86**	**26**

**Table 3:** Most used commercialized devices selected for this study, ordered by the manufacturer.

Manufacturer	#	Devices
Amazfit	16	Bip, Stratos, Bip S, Bip U, GTR, GTS, Stratos 3, GTR 2, GTS 2,
		T-Rex, GTR3, GTS 3, T-Rex 2, GTR 4, GTS 4, Falcon
Fitbit	11	Force, Charge, Surge, Blaze, Ionic, Versa, Versa 2, Versa 3,
		Sense, Versa 4, Sense 2
Apple	10	Watch 0, Watch Series 1, Watch Series 2, Watch Series 3, Watch Series 4,
		Watch Series 5, Watch Series 6, Watch Series 7, Watch Series 8, Watch Ultra
Samsung	8	Gear Live, Gear Fit 1, Gear Fit 2, Galaxy Watch 1, Galaxy Watch 2,
		Galaxy Watch 3, Galaxy Watch 4, Galaxy Watch 5
Garmin	7	Fenix 2, Fenix 3, Fenix 5, Fenix 6, Fenix 7, Venu, Instinct
Xiaomi	7	Smart Band 2, Smart Band 3, Smart Band 4, Smart Band 5,
		Smart Band 6, Smart Band 7, Watch
Philips	5	Health Watch, ActiWatch Spectrum, Biotel ePatch, BioSensor, Biotel MCOT
**TOTAL**	**64**	

**Table 4:** Journal publications selected for this study, ordered by publication year.

Year	#	References	Citations	Citations/Work
2013	3	[30,31,32]	155	51.67
2014	2	[33,34]	133	66.5
2015	6	[35,36,37,38,39,40]	287	47.83
2016	7	[41,42,43,44,45,46,47]	153	21.85
2017	4	[48,49,50,51]	136	34.0
2018	6	[52,53,54,55,56,57]	120	20.0
2019	6	[58,59,60,61,62,63]	125	20.83
2020	9	[64,65,66,67,68,69,70,71,72]	148	16.44
2021	15	[73,74,75,76,77,78,79,80,81,82,83,84,85,86,87]	99	6.6
2022	10	[88,89,90,91,92,93,94,95,96,97]	18	1.8
**TOTAL**	**68**	[30,31,32,33,34,35,36,37,38,39,40,41,42,43,44,45,46,47,48,49,50,51,52,53,54,55,56,57,58,59,60,61,62,63,64,65,66,67,68,69,70,71,72,73,74,75,76,77,78,79,80,81,82,83,84,85,86,87,88,89,90,91,92,93,94,95,96,97]	**1374**	**20.21**
